# New Insight into Inter-kingdom Communication: Horizontal Transfer of Mobile Small RNAs

**DOI:** 10.3389/fmicb.2017.00768

**Published:** 2017-05-01

**Authors:** Geyu Zhou, Yu Zhou, Xi Chen

**Affiliations:** State Key Laboratory of Pharmaceutical Biotechnology, NJU Advanced Institute for Life Sciences, Jiangsu Engineering Research Center for MicroRNA Biology and Biotechnology, School of Life Sciences, Nanjing UniversityNanjing, China

**Keywords:** inter-kingdom communication, small RNA, small interfering RNA, microRNA, mobile RNA

## Abstract

Small RNAs (sRNAs), including small interfering RNAs (siRNAs) and microRNAs (miRNAs), are conventionally regarded as critical molecular regulators of various intracellular processes. However, recent accumulating evidence indicates that sRNAs can be transferred within cells and tissues and even across species. In plants, nematodes and microbes, these mobile sRNAs can mediate inter-kingdom communication, environmental sensing, gene expression regulation, host-parasite defense and many other biological functions. Strikingly, a recent study by our group suggested that ingested plant miRNAs are transferred to blood, accumulate in tissues and regulate transcripts in consuming animals. While our and other independent groups’ subsequent studies further explored the emerging field of sRNA-mediated crosstalk between species, some groups reported negative results and questioned its general applicability. Thus, further studies carefully evaluating the horizontal transfer of exogenous sRNAs and its potential biological functions are urgently required. Here, we review the current state of knowledge in the field of the horizontal transfer of mobile sRNAs, suggest its future directions and key points for examination and discuss its potential mechanisms and application prospects in nutrition, agriculture and medicine.

## Introduction

Small non-coding RNAs (sRNAs), including small interfering RNAs (siRNAs) and microRNAs (miRNAs), are critical regulators of gene function (Carthew and Sontheimer, 2009). siRNA conventionally refers to exogenous double-stranded RNA (dsRNA) that is taken up by cells, while miRNA is single stranded and derived from endogenous stem-loop non-coding RNA (He and Hannon, 2004). Both siRNA and miRNA are processed inside the cell by the RNase Drosha or Dicer, bound by Argonaute (AGO) proteins and incorporated into the RNA-induced silencing complex (RISC), which, in most cases, negatively regulates target gene expression at the post-transcriptional level (He and Hannon, 2004; Carthew and Sontheimer, 2009).

The movement of sRNA molecules within an organism is a well-known phenomenon that facilitates gene silencing between cells and tissues in plants and nematodes (Sarkies and Miska, 2014). In higher mammals such as mice and humans, emerging evidence is accumulating that there is transfer of sRNAs, including siRNAs and miRNAs, between cells and tissues (Colombo et al., 2014). In 2008, it was first discovered that endogenous miRNAs are not restricted to the intracellular environment and are stably present and detectable in serum and plasma (Chen et al., 2008). The ultra-stability of miRNAs in the extracellular environment can be attributed to protection from RNase degradation by exosomes or RNA binding proteins (Chen et al., 2012). In fact, miRNAs are abundant and selectively packaged into exosomes (Valadi et al., 2007; Villarroya-Beltri et al., 2013), which can be delivered to another cell and function in mediating processes such as cell migration (Zhang et al., 2010), apoptosis (Guay et al., 2015), cancer metastasis (Zhou et al., 2014; Zhang et al., 2015), cell-to-cell interaction (Montecalvo et al., 2012), and immune response (Yin et al., 2014) in the new location. Exosomes can also be engineered to deliver exogenous siRNAs to pass through *in vivo* barriers and achieve specific gene silencing in target tissues with the potential for treating diseases such as neuronal degeneration disease (Alvarez-Erviti et al., 2011), morphine relapse (Liu et al., 2015) and cancer (Zhang et al., 2014).

On the other hand, it has also been noted that sRNA signals, mainly siRNA in the double-stranded form and miRNA in the single-stranded form, are transmitted between different species (Knip et al., 2014). Such cross-species transfer of sRNAs is a new form of crosstalk and communication between interacting or distantly related organisms (Knip et al., 2014). In this review, we summarize the latest findings regarding the cross-species transfer of sRNA molecules and focus on the mobility of sRNA from the perspective of trans-kingdom gene silencing.

## Double-Stranded siRNA Transfer Across Species

To date, the majority of examples of cross-species siRNA transfer come from interactions between host and parasite. Both animals and plants have been found to exchange double-stranded siRNAs with closely interacting pathogenic, parasitic or symbiotic organisms. The first report about siRNA traffic can be traced back to 1998, in which siRNAs were found to be taken up by *Caenorhabditis elegans* and blocked endogenous gene expression when *C. elegans* were fed a dsRNA-expressing bacterial strain (Timmons and Fire, 1998; Whangbo and Hunter, 2008). Since then, cross-species movement of siRNA silencing signals has been reported among a wide range of species. Plants can transfer double-stranded siRNAs to interacting organisms, such as fungi (Nowara et al., 2010; Koch et al., 2013), insects (Wu et al., 2016), and parasites (Westwood et al., 2009), to silence their transcripts and suppress their growth in a process referred as host-induced gene silencing (HIGS). Thus, the HIGS method provides us with a useful defense strategy for controlling invading pathogens and parasites. For example, transgenic plants engineered to produce dsRNAs against essential pest genes were more resistant to pest attack (Mao et al., 2007). On the other hand, the movement of siRNA molecules in the opposite direction has been reported (Weiberg et al., 2014, 2015). The plant pathogen *Botrytis cinerea* hijacks host RNAi pathways and exploits siRNAs to target defense genes in Arabidopsis and tomato, thereby enhancing its pathogenicity (Weiberg et al., 2013). These new findings indicate that mobile siRNAs should be added to the list of weapons in the host-parasite arms race.

## Single-Stranded miRNA Transfer Between Host and Invader

In addition to double-stranded siRNA, single-stranded miRNA was also found to be transferred between host and invader. miRNAs originating from parasites such as *Schistosoma japonicum* (Cheng et al., 2013; Hoy et al., 2014) and the nematode *Litomosoides sigmodontis* (Buck et al., 2014) have been found in the body fluids of infected individuals. Interestingly, miRNA signaling can be transmitted in the opposite direction from host to invader. For instance, LaMonte et al. (2012) found that sickle human erythrocytes utilize miRNAs to target *P. falciparum* genes and thereby defend against malaria. Notably, the translocated human miRNAs are not integrated into the canonical RNAi machinery but instead form chimeric fusions with *P. falciparum* transcripts, thereby inhibiting parasite translation (LaMonte et al., 2012). Liu et al. (2016) found fecal miRNAs can regulate bacterial transcripts and shape gut bacteria in mice. Plants (e.g., cotton) also export specific miRNAs to induce cross-kingdom gene silencing in pathogenic fungi and confer disease resistance (Zhang T. et al., 2016). Taken together, these studies suggest the transfer of single-stranded miRNA is widespread between host and parasites.

## Single-Stranded miRNA Transfer Between Animal Species

Despite the numerous cases in which the transfer of siRNA and miRNA silencing signals between different organisms has been described, these examples are predominantly found between hosts and their pathogens, parasites and symbionts. A highly debated issue that has not yet been resolved convincingly is whether there is the transfer of sRNAs between distantly related, complex organisms. Considering that humans consume a large amount of food (vegetables, fruits, and animal meats) every day, it is intriguing to speculate that exogenous sRNAs from distant species can enter our circulatory systems, stably accumulate to considerable doses and exert biological functions. Some studies focused on milk as a source of dietary animal miRNAs because a large fraction of miRNAs in milk are contained in exosomes and humans consume a large amount of milk annually. Baier et al. (2014) showed that plasma concentrations of miRNAs were significantly decreased when mice were fed with a milk miRNA-depleted diet for 4 weeks, suggesting that endogenous synthesis of miRNAs does not compensate for dietary miRNA deficiency. Consistent with these observations, emerging studies suggest that humans can absorb animal miRNAs from hard-boiled chicken eggs, including avian miRNAs that humans cannot synthesize (Zempleni et al., 2015). However, Title et al. (2015) and Auerbach et al. (2016) indicated that milk miRNAs are not taken up into murine offspring tissues or blood but are likely degraded by the digestive system.

## Can Single-Stranded miRNAs Transfer Across Kingdoms?

Because humans consume many plants every day, is it possible for different kingdom-derived molecules to achieve regulatory function? In 2012, our group published the first report that plant miRNAs ingested from food can pass through the gastrointestinal (GI) tract, enter into blood, accumulate in tissues and regulate endogenous gene expression in mammals (**Table 1**) (Zhang L. et al., 2012). In particular, we identified the presence of plant miR156a and miR168a in humans and mice by deep sequencing and quantitative RT-PCR. We further showed that, as miR168a is 2’-*O*-methylated, it must be diet-derived. To further confirm this hypothesis, we compared the serum miRNA profiles among mice fed with chow diet, a rice-based diet or chow diet supplemented with synthetic miR168a. We found miR168a to be robustly detected in mice fed with rice and miR168a-supplemented chow. Furthermore, we validated that miR168a can bind to and inhibit the translation of LDLRAP1. Because LDLRAP1 controls blood LDL levels, we further showed that mice fed with miR168a-enriched diets or rice had decreased levels of LDLRAP1 and increased levels of LDL. In summary, we were the first to demonstrate the accumulation and biological function of dietary miRNAs in animal tissues.

**Table 1:** Inter-kingdom transfer of animal and plant miRNAs.

Year	miRNAs	Source origin	Methods	Conclusion	Reference
2014	miR-29b, miR-200c	Milk	qRT-PCR	Positive	Baier et al., 2014
2015	miR-375	Milk	HTS, qRT-PCR, NB	Negative	Title et al., 2015
2016	miR-29b, miR-200c	Milk	OpenArray, qRT-PCR, HTS	Negative	Auerbach et al., 2016
2012	miR168a	Rice	HTS, qRT-PCR, NB, WB	Positive	Zhang L. et al., 2012
2012	miR168a	Plant	Mining public HTS datasets	Negative	Zhang Y. et al., 2012
2014	miR168a, miR156a, miR167a	Plant	Mining public HTS datasets	Negative	Tosar et al., 2014
2013	miR156a, miR159a, miR169a	Fruit	qRT-PCR	Negative	Snow et al., 2013
2013	miR156, miR160, miR166, miR167, miR168, miR172	Fruit	qRT-PCR, digital PCR	Negative	Witwer et al., 2013
2013	miR168a	Rice	HTS, qRT-PCR, ELISA	Negative	Dickinson et al., 2013
2016	Plant miRNAs	Plant	Computational prediction		Pirro et al., 2016
2015	Nine cow-milk miRNAs	Milk	Computational prediction, HTS	Positive	Shu et al., 2015
2016	Plant miRNAs	Plant	Computational prediction		Zhang H. et al., 2016
2015	Plant miRNAs	Soybean and rice	qRT-PCR	Positive	Philip et al., 2015
2015	miR2911	Honeysuckle	HTS, qRT-PCR, NB, Fluorescent labeled tracing assay	Positive	Zhou et al., 2015
2015	Many plant miRNAs	Fruit	qRT-PCR, NB	Positive	Liang et al., 2015
2014	miR172	Plant total RNA	qRT-PCR	Positive	Liang et al., 2014
2014	Plant miRNAs	Dietary food	HTS	Positive	Beatty et al., 2014
2016	miR166a, miR159	Bee pollen	HTS, qRT-PCR	Positive	Chen X. et al., 2016
2015	miR2911, miR168a	Honeysuckle decoction supplemented with synthetic MIR168a	qRT-PCR	Positive	Yang et al., 2015b
2015	miR2911	Dried herbs	qRT-PCR	Positive	Yang et al., 2015b
2016	miR2911	Vegetables	qRT-PCR	Positive	Yang et al., 2016
2016	miR159	Plant	qRT-PCR	Positive	Chin et al., 2016
2015	miR-34a	Synthesized methylated miRNA	qRT-PCR	Positive	Mlotshwa et al., 2015
2016	miR160, miR2673	*Brassica oleracea*	qRT-PCR	Positive	Pastrello et al., 2016
2016	Many mulberry miRNAs	Mulberry	TA clone, Sanger sequencing and droplet digital PCR		Jia et al., 2015
2017	Maize-derived miRNAs	Maize	qRT-PCR	Positive	Luo et al., 2017


*HTS, high-throughput sequencing; NB, Northern blot; WB, Western blot; ELISA, enzyme-linked immunosorbent assay*.

However, some follow-up studies generated contradictory results (**Table 1**). Two studies found that plant miRNAs were present in public animal sRNA datasets and suggested that these plant miRNAs could be contaminants during library preparation and sequencing (Zhang Y. et al., 2012; Tosar et al., 2014). Two other studies reported little or low measurable uptake of plant miRNAs by PCR in human and primates after a feeding study (Snow et al., 2013; Witwer et al., 2013). Dickenson et al. (2013) attempted to validate the original research (Zhang L. et al., 2012) but found little evidence of dietary uptake of miR168a after rice feeding and downregulation of LDLRAP1 by miR168a.

There are also a series of both computational and benchside studies from others and our group that support the dietary uptake of miRNAs (**Table 1**). Three studies provided theoretical solutions to address what type of plant miRNAs may be absorbed and whether there is any possibility for them to regulate endogenous gene expression. Pirro et al. (2016) developed a computational algorithm to search for functional sequence homologies between plant and mammalian miRNAs. The rationale behind their work is that, if possible, plant miRNAs must share homologous features with mammalian miRNAs to mediate cross-kingdom regulation. They successfully selected and experimentally validated the prediction that miR168a can regulate the expression of SIRT1 in human cell lines. Another study focused on selecting and comparing the structural and sequence features of miRNAs and predicted the likelihood of transportation based on classification (Shu et al., 2015). They further showed that miRNAs predicted to be highly transportable share similar sequences with their homologs in humans and are likely associated with exosomes and further validated the presence of nine cow’s milk miRNAs in human plasma (Shu et al., 2015). Another work by Zhang H. et al. (2016) found through network analysis that the reported ingested plant miRNAs may have a functional impact on consuming organisms.

In addition to bioinformatics studies, many experimental studies have provided direct evidences for the absorption and active function of cross-species plant miRNAs (**Table 1**). Philip et al. (2015) confirmed that dietary plant miRNAs are stably present in intact form after storage, processing, cooking and early digestion in a simulation study. This study potentially indicated that plant miRNAs are robust and bioavailable for uptake during early digestion *in vivo*. Another work by Luo et al. (2017) suggests that maize-derived miRNAs could be detected in porcine tissues and serum. Our follow-up study identified miR2911, a honeysuckle (HS)-encoded atypical miRNA, as the most stable miRNA in HS decoction after the boiling process. Since HS is a well-known Chinese herb used for the treatment of influenza A virus (IAV) infection, we tested if miR2911 is capable of exerting a therapeutic effect on IAVs. We showed that continuous drinking or gavage feeding of the HS decoction led to significant elevation of miR2911 in mouse blood and lung. As a consequence, miR2911 in the HS decoction directly suppressed various IAVs, including H1N1, H5N1, and H7N9, both *in vitro* and *in vivo* (Zhou et al., 2015). Our group also conducted a kinetics study to show that the absorption of plant miRNA is a real physiological event, since a technical artifact or contamination is unlikely to form a dynamic pattern and typical kinetic absorption curve (Liang et al., 2015). Additionally, an independent group reported that foreign plant miRNAs could be detected in the sera, feces, and several tissues after feeding mice plant total RNAs: small RNAs (20–100 nt) can survive for 36 h or longer in blood and fecal samples; the level of miR172 recovered from the stomach of one mouse reached a maximum of 4.5%, with a range of 0.05–4.5% in different organs (Liang et al., 2014). Beatty et al. (2014) identified abundant non-human small RNA sequences in plasma and plasma exosomal samples, a small portion of which were derived from dietary plant material. Interestingly, they found that the plasma sample obtained from an individual who reported following a vegetarian diet had a relatively high proportion of plant sequences. Furthermore, a study identified exogenous miRNAs from rapeseed bee pollen in mouse blood after feeding (Chen X. et al., 2016). Yang et al. (2015b) were able to detect miR2911 and miR168a from the sera of mice fed a chow diet containing HS and synthetic miR168a. The plant miRNA levels decreased to background levels after the HS diet was replaced with a normal chow diet, proving that the detected miRNAs are absorbed from food (Yang et al., 2015b). Further work by Yang et al. (2015a, 2016) suggested that miR2911 exhibited unusual stability, was not associated with exosomes or the Argonaute complex during circulation and that the stability may be conferred by modifications from the host. They also showed that unlike most of plant derived biomolecules, miR2911 is atypical as their amount is positively correlated with the degradation of plant food and rRNAs and their biogenesis is Dicer independent (Yang et al., 2017). Chin et al. (2016) confirmed that plant miR159 was predominantly detected in extracellular vesicles in Western human sera, with the abundance of this miRNA in the serum being inversely correlated with breast cancer incidence and progression in patients. They further showed that oral administration of a miR159 mimic significantly suppressed the growth of xenograft breast tumors in mice by targeting TCF7, which encodes a Wnt signaling transcription factor, leading to a decrease in MYC protein levels (Chin et al., 2016). The immunoregulatory effect of strawberry miRNAs has recently be explored as they can function as ligands and bind to TLR3 on dendritic cells and modulate their response to inflammatory agents (Cavalieri et al., 2016). Interestingly, scientists have taken advantage of food-derived miRNAs in mammals and engineered crop plants to express artificial miRNAs that can silence essential human genes to treat cancer. Mlotshwa et al. (2015) confirmed that tumor suppressor miRNAs designed to mimic small RNAs produced in plants were taken up by the digestive tract of Apc^Min/+^ mice upon ingestion, as indicated by their higher concentration in the miRNA-treated animals and the consequent reduction in tumor burden. Pastrello et al. (2016) confirmed the presence of plant miRNAs in human blood and suggested that miRNAs cooperate with other Brassica-specific compounds in a possible cancer-preventive mechanism. Interestingly, this inter-kingdom crosstalk is not limited to plants and mammals. Jia et al. (2015) used silkworm and mulberry as a model for investigating the interactions between insect and plant via miRNA-mediated cross-kingdom communication. They carefully conducted multiple assays, including TA clones, Sanger sequencing and droplet digital PCR, to eliminate the possibility of contamination as proposed by others in library preparation for next generation sequencing (Zhang Y. et al., 2012; Tosar et al., 2014) and confirmed that mulberry-derived miRNAs can enter silkworm hemolymph and multiple tested tissues. Unfortunately, as feeding silkworms synthetic miR166b did not cause any phenotypic changes, they concluded that the ingestion of plant miRNAs by silkworms is not functional. Nevertheless, this study suggests that inter-kingdom plant miRNA transfer may be a conserved phenomenon, but its influence on the physiological progress of recipient animal organism remains to be elucidated.

## Key Points for Examination

It is acknowledged that reproducibility is the gold standard for evaluating a research finding. Thus, we think it is highly important to carefully investigate and validate original research. Meanwhile, it is not advisable to make a perfunctory refutation based on one experiment or limited evidence. Cross-validation is required to draw a scientific conclusion. For instance, as many studies have reported the possibility of contamination and false positives, we generally followed three steps to exclude the possibility of technical artifacts and to ensure the detected plant miRNAs are real: (a) High-throughput sequencing: we only selected plant miRNAs with high sequencing reads (*N* > 100 copies after normalization), and the miRNAs with high reads likely are real; (b) qRT-PCR assay: this assay, with a standard curve, a no-template reaction serving as a control, and a proper inner standard and RNA extraction method, was used to cross validate high-throughput sequencing results; and (c) northern blot analysis. It is highly unlikely that plant miRNAs identified by three different techniques are derived from contamination or technical artifacts. With these three different assay platforms, we believe that the number of “false signals” can be reduced to a minimum.

Second, some studies ignored the critical fact that the conventional protocols and solutions in the sequencing procedure are designed to enhance the precision and efficiency of identifying animal miRNAs (Chen et al., 2013). As the 2′-*O*-methyl modification of the 3′-ends of plant miRNAs results in decreased adaptor ligation efficiency (Munafo and Robb, 2010), the sequencing procedure is biased against plant miRNAs compared with non-modified animal miRNAs. In other words, if a sample contains a mixture of 2′-*O*-methyl plant miRNA and 2′-OH animal miRNA, ligations would favor the capture of the 2′-OH animal miRNA. It is therefore not surprising that the sequencing reads of plant miRNAs are masked in the vast ocean of animal miRNAs during the sequencing of mixed plant and animal libraries, which can explain the negative results of some studies.

Third, we realized that several studies reported the failure to efficiently detect exogenous miRNAs after performing the feeding experiments. We believe there are several factors causing this phenomenon. First, the concentration of miRNAs should be within the linear working range of qRT-PCR. If the level of miRNA is too low, it would fall outside the working range of qRT-PCR, and no signals would be effectively detected. Thus, the absolute quantification method rather than the relative quantification method should be employed to determine the levels of plant miRNAs in animal tissues and blood. It is also essential to include the experimental details about raw CT values, background signals and non-specific amplification products; otherwise, the robustness of the results is difficult to evaluate. Second, an internal control or reference gene should be employed in the qRT-PCR assay to normalize exogenous plant miRNAs. Proper normalization is critical for quantitative analysis of miRNAs, as variations in the amount of material, sample collection, RNA extraction and enzymatic efficiency can introduce potential bias and contribute to quantification errors. Third, the process of extracting RNA from tissues or blood greatly affects the outcome of the quantitative detection of plant miRNAs. Therefore, a suitable RNA isolation method must be selected to accurately detect plant miRNAs in animal tissues or blood, since the detection of exogenous plant miRNAs in animal tissues/blood lies at the foundation of exploring their cross-kingdom regulatory functions. By serving as a guideline, these three points may clarify the controversial issues in the quantification of exogenous plant miRNAs in the tissues and blood of humans and other animals.

## Potential Mechanisms and Future Directions

Despite many observations of inter-kingdom sRNA transfer, several mechanistic aspects of this signaling process remain largely unknown. The first crucial point is identifying how much sRNA within the body is functional. Because mammals do not possess an amplification pathway similar to *C. elegans* to allow a small amount of RNA taken up from the environment to generate abundant secondary RNAs and trigger an extensive response within the body, the sRNA itself would have to be taken up in sufficient amounts to have an effect. Since sRNA is enriched in the everyday food of human beings, foreign sRNA might survive the trip to the intestine and be absorbed by the body through sufficient dietary intake. In addition, there are many challenges that sRNAs must face as they are taken up by the body via food sources before reaching their target organs. For example, once inside the mammalian GI tract, exogenous sRNAs face a number of extreme factors, including RNases, phagocytosis and a low-pH environment. These unfavorable conditions require sRNAs to adopt stable structures to protect themselves from degradation prior to reaching recipient cells. Because plant miRNAs are methylated on the 3′-terminal nucleotide and are relatively resistant to exonuclease digestion, methylation may be a mechanism accounting for the stability of plant miRNAs *in vivo*. Consistently, we detected that mature plant miRNAs have slower degradation rates than synthetic unmethylated miRNAs (Zhang L. et al., 2012). Several studies have also observed that certain plant miRNAs are resistant to extreme environments, such as boiling and early digestion (Philip et al., 2015; Zhou et al., 2015).

Another key mystery is how sRNAs pass through the GI tract and recognize and enter their target cells. Recently Luo et al. (2017) used an *ex vivo* everted gut sac model to simulate the real physiological condition and supported the assertion that exogenous miRNA can be absorbed in the intestine. In *C. elegans*, the spreading of RNAi effects to cells and tissues that have not initially encountered a dsRNA trigger is observed in a phenomenon called systemic RNAi (Timmons and Fire, 1998). SID-1, a transmembrane protein belonging to the systemic RNA interference defective (SID) family, was identified to be required for systemic RNAi following a screen of *C. elegans* mutants lacking the wild-type systemic RNAi phenotype (Winston et al., 2002; Feinberg and Hunter, 2003). Now, it is generally accepted that systemic RNAi, mediated mainly through SID-1, allows small RNAs to be transported across cellular boundaries and to subsequently spread throughout the whole body of many organisms. However, since systemic RNAi has only been intensively characterized in insects and is unlikely to exist in mammals, it is still important to identify the channel protein, carrier or transporter that is responsible for sRNA uptake and traffic. Whether an SID-1 homolog is present in mammals and plays an equal role in small RNA transport requires further investigation. On the other hand, because every biological barrier has some degree of leakiness, Yang et al. (2015c) proposed a model suggesting that the dietary consumption of HS facilitates the uptake of miR2911 by affecting the permeability of the GI tract or influencing the microbiome within the GI tract.

How exogenous sRNAs travel within the host is another important issue deserving further discussion. One possible mechanism is that sRNA traveling between organisms relies on mechanisms similar to those observed for extracellular transport within an organism. In mammals, cell-to-cell communication is mediated by extracellular exosomes that contain miRNAs (Mittelbrunn and Sanchez-Madrid, 2012). Therefore, it is interesting to speculate that the vesicular uptake of sRNA on one side of a biological barrier and release on the other side is precisely the mechanism facilitating exogenous sRNA transfer. Because the majority of plant miRNAs detected in animal serum were found inside exosomes (Zhang L. et al., 2012), we hypothesize that intestinal epithelial cells are able to take up plant miRNAs and package them into exosomes, and the plant miRNAs are subsequently delivered to recipient tissues and cells via the endogenous exosome circulatory system. In agreement with this, it has been observed that exosomes secreted by the GI nematode *Heligmosomoides polygyrus* transfer miRNAs to mammalian cells and modulate innate immunity (Buck et al., 2014). On the other hand, a previously unrecognized strategy by which plants communicate with mammalian cells in the gut was recently revealed. Mu et al. (2014) characterized edible plant-derived exosome-like nanoparticles (EPDENs) and showed that they possess a similar size and structure to mammalian exosomes and contain proteins, lipids and miRNAs. They also showed that EPDENs are orally taken up by intestinal macrophages and stem cells and have biological effects on the recipient cells. Remarkably, this finding opens up a new avenue to further study the molecular mechanisms underlying EPDEN-mediated crosstalk between the plant kingdom and mammalian cells.

Exogenous sRNAs mainly regulate gene expression in the conventional RNAi manner although studies have found that they can also act as competitive ligands against inflammatory agents (Cavalieri et al., 2016). However, it is unclear how exogenous sRNAs are incorporated into the host Argonaute complexes and use their RNAi machinery to convey the silencing effect. To be loaded into Argonaute proteins to function, it is generally thought that the dsRNA form is required. However, the ingested plant miRNAs should be the mature single-strand RNA form. Actually, there are exceptions. For example, single-stranded priRNAs from the degradation products of abundant structural RNAs (e.g., rRNA and tRNA) can be loaded directly onto AGO1 in fission yeast (Halic and Moazed, 2010; Jain et al., 2016). Another example in mammals is that single-stranded tRNA fragments were well characterized to associate with Argonautes (Kumar et al., 2014; Chen Q. et al., 2016; Sharma U. et al., 2016), although this loading is less understood. Because the hijacking of host cell RNAi machinery by miRNAs to exert biological functions has been frequently detected (Nowara et al., 2010; Helber et al., 2011; Liu et al., 2012; Koch et al., 2013; Weiberg et al., 2013; Ghag et al., 2014), it is therefore interesting to analyze the mode of action of plant miRNAs in host cells.

## Significance and Conclusion

Inter-kingdom sRNA crosstalk may be universal and involves complex organisms such as plants and animals. This emerging field may impact agriculture, nutrition, pharmacology, and medical research. First, the inter-kingdom transfer of sRNA will be a valuable tool for future use in the development of crop protection. Engineering crops to express anti-fungal or anti-pest dsRNA can render them resistant and thus likely increase food production. Second, inter-kingdom sRNA transfer opens the field of identifying the nutritional value of dietary sRNAs, such as whether dietary miRNAs present in milk will have a biological impact on consumers. Last but not least, plants seem to be ideal delivery carriers for therapeutic sRNAs, as some plant miRNAs are robust and resistant to the harsh conditions during digestion. For example, it has been identified and confirmed that natural or artificial plant miRNAs can inhibit the expression of disease-related genes both *in vitro* and *in vivo* (Zhou et al., 2015; Chin et al., 2016; Sharma A. et al., 2016; Tian et al., 2016; Choi et al., 2017). This opens a potential novel avenue for engineering transgenic plants to become medicinal plants that will produce and help deliver therapeutic plant miRNAs, such as miR2911 to treat influenza and miR159 to treat breast cancer, to consumers.

## Author Contributions

GZ and XC wrote the manuscript. YZ edited the manuscript.

## Conflict of Interest Statement

The authors declare that the research was conducted in the absence of any commercial or financial relationships that could be construed as a potential conflict of interest.
